# Controversies in NEN: An ENETS position statement on the treatment of patients with Grade 3 well‐differentiated neuroendocrine tumours of the gastro‐enteropancreatic tract

**DOI:** 10.1111/jne.70080

**Published:** 2025-08-09

**Authors:** Mairéad G. McNamara, Halfdan Sorbye, Nehara Begum, Emanuel Christ, Nicola Fazio, Lynnette Fernandez‐Cuesta, Rocio Garcia‐Carbonero, Gregory Kaltsas, Atsuko Kasajima, Ramon Salazar, Ernst Jan M. Speel, Andreas Kjaer

**Affiliations:** ^1^ Division of Cancer Sciences University of Manchester Manchester UK; ^2^ Department of Medical Oncology The Christie NHS Foundation Trust Manchester UK; ^3^ Cancer Clinic Haukeland University Hospital Bergen Norway; ^4^ Department of Clinical Science University of Bergen Bergen Norway; ^5^ Department for General‐, Visceral‐, Thoracic‐ and Endocrine Surgery, ENETS‐Center of Excellence, Johannes‐Wesling‐Klinikum Minden University Hospital of the Ruhr‐University Bochum Minden Germany; ^6^ Division of Endocrinology, Diabetology and Metabolism University Hospital of Basel Basel Switzerland; ^7^ Division of Gastrointestinal Medical Oncology and Neuroendocrine Tumors IEO IRCCS, European Institute of Oncology Milano Italy; ^8^ Computational Cancer Genomics Team, Genomic Epidemiology Branch International Agency for Research on Cancer (IARC‐WHO) Lyon France; ^9^ Medical Oncology Department, Hospital Universitario 12 de Octubre, IIS Imas12, Facultad de Medicina Universidad Complutense de Madrid (UCM) Madrid Spain; ^10^ 1st Propaedeutic Department of Internal Medicine National and Kapodistrian University of Athens Athens Greece; ^11^ Department of Pathology, TUM School of Medicine and Health Technical University Munich Munich Germany; ^12^ Medical Oncology Department Institut Català d'Oncologia. Oncobell Program (IDIBELL), Universitat de Barcelona (Campus Bellvitge), CIBERONC Barcelona Spain; ^13^ Department of Pathology and Clinical Bioinformatics Erasmus Medical Center Rotterdam The Netherlands; ^14^ Department of Clinical Physiology and Nuclear Medicine Copenhagen University Hospital – Rigshospitalet Copenhagen Denmark; ^15^ Cluster for Molecular Imaging, Department of Biomedical Sciences University of Copenhagen Copenhagen Denmark

**Keywords:** well‐differentiated NET, digestive, Grade 3, NET G3, neuroendocrine neoplasm

## Abstract

Grade 3 neuroendocrine tumours (NET G3) represent approximately 20% of high‐grade neuroendocrine neoplasms, and the recent identification of this entity has given rise to many unanswered questions relating to clinical management. The prognosis for these patients is worse than for those with Grade 1–2 well‐differentiated NET, but better than for those with Grade 3 poorly differentiated neuroendocrine carcinoma. This consensus statement aims to address some uncertainties and explore unmet needs in the management of patients with NET G3. Firstly, the role of surgery in localised disease will be discussed as well as the dilemma in relation to the use of neo‐adjuvant and/or adjuvant treatment in this setting. Treatment of oligometastatic digestive NET G3 will also be examined, including the positioning of surgery and ablative therapy. In the advanced setting, traditionally, chemotherapy in the form of temozolomide/capecitabine or 5‐fluorouracil‐based therapies, rather than platinum/etoposide, is considered a first‐line treatment option, with second‐line therapy dependent on what was used first‐line. More recently, following the results of the NETTER‐2 trial, Peptide Receptor Radionuclide Therapy with ^177^Lu‐DOTATATE may be an option for selected patients with somatostatin receptor positive NET G3. There is limited data on the use of immunotherapy and targeted therapy in this disease group to date, and some available evidence will be presented. The role for re‐biopsy to guide treatment decision‐making in patients with digestive NET G3 and whether NET G3 outside of the digestive tract should be treated similarly will also be scrutinised. Prospective studies with translational end‐points are required to enable a better understanding of this diagnosis and to facilitate more optimal treatment discoveries.

## 1 INTRODUCTION

After the release of the 2010 *World Health Organization* (WHO) classification, it was recognised that the neuroendocrine tumour (NET) category included well‐differentiated tumours with a Ki‐67 greater than 20% and therefore overlapped with the Ki‐67 range of neuroendocrine carcinoma (NEC), and exhibited a more aggressive course than Grade 1–2 (G 1–2) NET. In 2017, the WHO classification of pancreatic tumours thus added a NET G3 category. Consequently, two high‐grade neoplasms were identified, NET G3 and NEC.^1^ The distinction between the two was based on well‐differentiated and poorly differentiated histology and genetic profiles.^2^ While NET G3 generally retains the organoid pattern and wild‐type TP53 and Rb1 gene profiles of well‐differentiated neuroendocrine neoplasms (NENs), NEC usually shows a disorganised pattern, significant cellular atypia, and genetic abnormalities that resemble their exocrine counterparts, including *TP53, KRAS, BRAF*, or *Rb1* genes, among others.^2^, ^3^

Although initially it was thought that the majority of NET G3 arose from the pancreas, subsequent studies^4^ showed that these NETs could also occur in other organs, such as small bowel, lung, stomach, and rectum.^5^ Rarely, NET G3 are found in the duodenum and ampulla of Vater, whereas the appendix and oesophagus do not seem to be involved.^5^ It has been reported that NET G3 comprise approximately 18%–20% of NEN G3.^6^, ^7^

Clinically, the majority of NET G3 have metastases at diagnosis (predominantly in the liver), most commonly originating from the pancreas (42%), followed by the lung (9%), ileum (7%), stomach (3%), rectum (1%), and rare sites (2%) such as the prostate and breast. In 15%–24%, the primary site is unknown.^5^, ^7^ NET G3 express somatostatin receptor 2 (SSTR2) in 70%–85%, which is comparable to that of NET G1/G2 and significantly higher than that of NEC.^5^, ^7^, ^8^

The evolution of NET G3 is incompletely understood, but is clinically important as the prognosis for patients with NET G3 is worse than for those with NET G1/G2, but better than for NEC.^9^ Recently, it has also been reported that patients with NET G3 with a Ki‐67 <55% have a shorter overall survival (OS) if treated with platinum‐based treatment versus temozolomide‐based treatment if they have a poor performance status, are older, and have elevated alkaline phosphatase levels.^7^ It is also evident that the Ki‐67 value increases synchronously and metachronously in metastases of digestive NETs and correlates with poorer patient outcomes.^10^ The observed increase in Ki‐67 and higher histological atypia during disease progression suggests that NET G3 may arise from lower grade NET.^11^, ^12^, ^13^, ^14^, ^15^ The evolution from NET G3 to NEC has been proposed, but recently questioned in studies on sequential biopsies from liver metastases of patients with NET G3 undergoing treatment.^12^, ^13^ In one study, there was a sharp increase in Ki‐67 that was associated with *TP53* mutations and focally NEC‐like histological transformation in 22% of patients with NET G3 followed for a median of 29 months. However, a complete transformation to a typical NEC was not observed.^13^ There was also no clear correlation with the therapies given, but recent work has postulated that specific therapeutic regimens may be associated with an abrupt transformation of a NET to high‐grade NEN.^14^, ^16^

There is substantial uncertainty regarding the therapeutic pathways for patients with a digestive (including pancreas) NET G3 diagnosis, and the following consensus statement aims to answer some identified priority questions (Table 1) by critically evaluating the currently available data.

**TABLE 1:** Controversies surrounding the therapeutic pathways for patients with a digestive (including pancreas) Grade 3 well‐differentiated NET diagnosis.

Controversy	Questions
(1) Peri‐operative treatment of digestive NET G3	(1a) Should surgery be considered for localised digestive NET G3? (1b) What is the role of neo‐adjuvant therapy in potentially resectable digestive NET G3? (1c) Is there a role for adjuvant treatment for resected digestive NET G3?
(2) Treatment of oligometastatic digestive NET G3	(2a) Should surgery be considered for oligometastatic digestive NET G3? (2b) What is the role of local ablative therapies in oligometastatic digestive NET G3?
(3) Treatment of advanced disease (systemic treatment including PRRT)	(3a) What treatment should be given to patients with digestive NET G3 in the first‐line advanced setting? (3b) What treatment should be given to patients with digestive NET G3 in the post first‐line advanced setting?
(4) Treatment of advanced disease with immunotherapy	(4a) Is there a role for immunotherapy in the treatment of patients with advanced digestive NET G3?
(5) Treatment of advanced disease with targeted therapy	(5a) Is there a role for targeted therapy in the treatment of patients with advanced digestive NET G3?
(6) Other controversies	(6a) Is there a need for re‐biopsy to guide treatment decision making in patients with digestive Grade 1–Grade 3 well‐differentiated NET? (6b) Is NET G3 outside of the digestive tract treated similarly?

Abbreviations: G3, Grade 3; NET, neuroendocrine tumour; PRRT, peptide receptor radionuclide therapy.

## 2 CONTROVERSIES

1. Peri‐operative treatment of digestive NET G3:

1a. Should surgery be considered for localised digestive NET G3?
1b. What is the role of neo‐adjuvant therapy in potentially resectable digestive NET G3?
1c. Is there a role for adjuvant treatment for resected digestive NET G3?

### Current evidence

Surgical resection is currently the mainstay of therapy for patients with localised digestive NET G3,^17^ with 3‐ and 5‐year recurrence‐free survival (RFS) rates of 49.1% and 24.5%, respectively, and a median OS of approximately 50 months.^18^, ^19^ The *European Neuroendocrine Tumor Society* (ENETS) guidelines advocate for complete resection in patients with G1 and G2 well‐differentiated gastro‐enteropancreatic NET when feasible, as surgery offers the only potential for cure in localised disease.^20^ Retrospective analyses indicate that despite the higher proliferative index associated with G3 tumours, well‐differentiated morphology may confer a survival benefit with curative‐intent surgery. For instance, studies suggest that a subset of patients with localised NET G3 can achieve prolonged disease‐free intervals following radical resection.^19^, ^21^, ^22^ A retrospective study, which included a limited number of patients with surgically resected G3 pancreatic NEN, reported that post‐operative survival was similar for those with NET G3 (*N* = 13) and NEC (*N* = 15); therefore, they concluded that surgical intervention for high‐grade tumours should be considered judiciously, acknowledging the small sample size.^23^

Prospective studies in the neoadjuvant setting are lacking. A neoadjuvant approach is especially relevant for patients with an initial non‐resectable primary, which can be resected if sufficient tumour shrinkage is achieved. In retrospective reports in patients with advanced digestive NET G3, response rates (RR) of 30%–40% have been reported after treatment with capecitabine combined with temozolomide (CAPTEM) or 5‐fluorouracil/oxaliplatin (FOLFOX), and these regimens may be neoadjuvant options to consider when treating localised tumours that are initially unresectable.^24^, ^25^, ^26^, ^27^, ^28^, ^29^, ^30^, ^31^ There are also emerging data from retrospective studies in high‐grade NEN evaluating other multimodal approaches, including the neoadjuvant chemotherapy regimen modified 5‐fluorouracil/irinotecan/oxaliplatin (mFOLFIRINOX) which has demonstrated meaningful RRs that may downstage tumours, rendering initially borderline resectable lesions operable.^32^, ^33^ However, the number of patients included with NET G3 is small. A study also investigated the reduction rate over time of sunitinib in 60 patients (10 with NET G3) with unresectable or distant metastatic pancreatic NEN;^34^ a median maximum reduction rate of 18.3% was reported.^34^ Neoadjuvant treatment with Peptide Receptor Radionuclide Therapy (PRRT) may be another option to downsize an initial localised non‐resectable somatostatin receptor imaging (SRI)‐positive primary tumour, as the RR to PRRT for the NET G3 subgroup in NETTER‐2 was as high as 48%.^35^, ^36^ Although these results are preliminary and the numbers of patients included with NET G3 were limited, they support further investigation into neoadjuvant strategies, particularly for locally advanced tumours and tumours with high‐risk features (e.g., elevated Ki‐67).

### Expert consensus

Expert panels acknowledge that while upfront surgery is recommended for localised NET G3, the role of neoadjuvant therapy is best reserved for patients with tumours demonstrating aggressive features or marginal resectability.^37^ In these cases, a multimodal approach may reduce tumour burden and improve resectability, including neoadjuvant chemotherapy or PRRT for SRI‐positive tumours, although controversy remains as the evidence is not yet definitive.^38^ Similarly, the role of adjuvant therapy after surgery is controversial. Some experts propose that adjuvant treatment (systemic chemotherapy) might benefit patients at high risk, particularly those with borderline resection margins or lymphovascular invasion.^39^ Yet, current ENETS recommendations stress that there is insufficient evidence to standardise adjuvant therapy in this setting.^21^, ^40^

### Patient selection criteria

Key factors influencing treatment decisions include patient‐related factors (age, *Eastern Cooperative Oncology Group Performance Status* (ECOG PS), co‐morbidities) and tumour‐related features (location, Ki‐67 proliferation index, and imaging findings that assess resectability). Cases should be evaluated by a multidisciplinary team. Comprehensive staging and functional imaging (e.g., dual somatostatin receptor/^18^F‐Fluorodeoxyglucose Positron Emission Tomography/Computed Tomography (^18^F‐FDG PET/CT)) are essential to tailor peri‐operative management. Selection for neoadjuvant therapy should focus on cases with borderline resectability or where tumour biology (such as a rapid doubling time) suggests an aggressive course of disease.^41^

### Conclusion and clinical recommendations

In summary, surgery should be considered in patients with localised digestive NET G3, with complete resection offering the best chance for long‐term survival. Neoadjuvant therapy may be beneficial for patients with borderline resectable tumours, although current evidence is limited to retrospective studies and expert consensus. Adjuvant treatment remains investigational, with decisions best made on a case‐by‐case basis after multidisciplinary review. Table 2 details some listed adjuvant studies on *clinicaltrials.gov* including patients with digestive NET G3. Randomised trials are needed to clarify the roles of neoadjuvant and adjuvant therapies and to determine optimal sequencing in the perioperative management of these tumours.

2 Treatment of oligometastatic digestive NET G3:

2a. Should surgery be considered for oligometastatic digestive NET G3?
2b. What is the role of local ablative therapies in oligometastatic digestive NET G3?

**TABLE 2:** Selected listed adjuvant studies on clinicaltrials.gov including patients with digestive Grade 3 well‐differentiated neuroendocrine tumours.

NCT number	Phase	Population	Planned enrolment	Primary end‐point(s)	Location
04166006	Single arm phase 2	Adjuvant vaccination with dendritic cells loaded with autologous tumour homogenate in resected stage 4 rare cancers (head & neck, NET & soft tissue sarcoma)	51	Incidence of treatment‐emergent adverse events & immunological efficacy	Italy
06158516	Randomised phase 2	Resected pancreatic NET (Grade 1–3 with >/= 1 high‐risk postoperative recurrence factor^a^) (surufatinib versus placebo post‐surgery)	100	2‐year disease‐free survival	China
05040360	Randomised phase 2	Resected well‐differentiated pancreatic NET (Ki‐67 >/=3% to </= 55%) (Capecitabine/Temozolomide up to 4 cycles versus surveillance)	141	Recurrence‐free survival	USA

Abbreviations: NET, neuroendocrine tumour, USA, United States of America.

^a^ Lymph node metastasis, neurovascular invasion, pancreatic duct dilatation, tumour > 4 cm, positive resection margin, or Grade 1 with lymph node involvement, Clinicaltrials.gov last accessed on July 2nd 2025.

### Current evidence

The management of oligometastatic (spread to fewer than five sites in the body) digestive NET G3 requires an individualised approach. Patients with resectable metastases should be considered for surgery with radical intent, as this may provide long‐term disease control in some patients. Other locally ablative therapies may also be considered for certain anatomic sites, though specific evidence in the context of NET G3 is lacking. Recent literature suggests that select patients with limited metastatic burden may benefit from surgery plus/minus other loco‐regional treatment modalities, which can prolong progression‐free survival (PFS) and potentially improve OS. In 27 patients with NET G3 with liver metastases, hepatic resection/ablation (48% complete cytoreduction/89% with >90% cytoreduction) resulted in a median PFS of 0.8 years and a median OS of 6.3 years, with some patients having long‐term survival.^42^ Although data remain retrospective, ENETS guidelines support the use of surgery in highly selected cases with oligometastatic disease, emphasising the importance of achieving maximal cytoreduction.^21^, ^43^ While large‐scale randomised trials are lacking, institutional series have provided insight into multimodal management.^44^ For instance, emerging evidence from studies employing transarterial embolisation (TAE) or transarterial chemoembolisation (TACE) for liver metastases of gastro‐enteropancreatic NET (G3, *N* = 19 (7.1%)) has shown encouraging local control rates in patients with predominantly liver disease and a limited number of metastases.^45^ Stereotactic body radiotherapy may also be an option for local control in patients not fit for surgery.^46^

### Expert consensus

Multidisciplinary consensus underscores that aggressive local treatment, including surgery and local ablative therapies, could be considered for patients with oligometastatic NET G3 who have a favourable performance status following multidisciplinary team meeting discussion. Patient selection is critical; candidates should typically have low‐volume liver metastases with controlled primary disease. Expert opinion further highlights that, in these settings, cytoreductive surgery may be combined with local ablative modalities to maximise disease control.^43^

### Patient selection criteria

Patients with oligometastatic disease should be rigorously evaluated using functional imaging (SRI PET/CT, FDG‐PET/CT)^47^ and cross‐sectional modalities to assess the extent of metastatic spread. Dual PET imaging with SRI and FDG‐PET can help to identify well‐differentiated and less differentiated tumour areas, enabling a tailored approach. Criteria include a limited number of metastatic lesions (often confined to the liver), a high uptake on SRI, and well‐preserved organ function. The decision for local therapies should be made in a multidisciplinary setting involving surgical, medical, and interventional radiology experts.^31^, ^48^

### Conclusion and clinical recommendations

For patients with oligometastatic digestive NET G3, surgery could be considered in select cases, particularly when a complete or near‐complete cytoreduction is achievable. Local ablative therapies offer promising alternatives or adjuncts in patients unsuitable for surgery or with multifocal, but limited disease.^49^ A personalised treatment plan, guided by multidisciplinary discussion and rigorous imaging assessment, is essential. Further prospective studies are needed to better define the survival benefit and optimal sequencing of these local interventions.

3 Treatment of advanced disease (systemic treatment, including PRRT):

3a. What treatment should be given to patients with digestive NET G3 in the first‐line advanced setting?

The optimal first‐line systemic treatment of advanced NET G3 is not well established. A NET G2‐like treatment strategy is frequently used; however, NET G3 is more aggressive, with shorter survival than NET G2. Available data support the use of PRRT or chemotherapy (e.g., CAPTEM or FOLFOX) as first‐line treatment for advanced disease. Based on the effect in NET G1‐2, streptozotocin/5‐fluorouracil (5‐FU) could be considered when the primary is pancreatic, but specific data for NET G3 are limited.^50^ First‐line use of platinum/etoposide results in a shorter OS for NET G3 with a Ki‐67 < 55%.^7^ NET G3 usually has high uptake on ^18^F‐FDG PET/CT and may also have uptake on SRI. Dual imaging is recommended to prevent PRRT from being delivered to patients with discordant (FDG positive, SRI negative) lesions.

### PRRT

The NETTER‐2 study is the only reported randomised PRRT trial with inclusion of patients with a NET G3 diagnosis.^35^ Patients with advanced gastro‐enteropancreatic NET with high, homogeneous SRI uptake and a Ki‐67 of 10%–55% were randomised to first‐line treatment with ^177^Lu‐DOTATATE or high‐dose octreotide LAR. Recently, the subgroup analyses for NET G3 have been presented.^36^ The RR for NET G3 was 48.1% and PFS 22.2 months with ^177^Lu‐DOTATATE (*n* = 52) vs. 7.4% and 5.6 months for high‐dose somatostatin analogues (SSA), respectively (*n* = 27). The NETTER‐2 trial clearly demonstrated clinically meaningful activity and significant PFS superiority of PRRT versus high‐dose SSA (control arm: 60 mg octreotide intramuscularly every 4 weeks in the first‐line setting) as a first‐line treatment option for patients with NET G3 SRI‐positive tumours and Ki‐67 <55%.

### Medical treatment

In retrospective reports in patients with advanced digestive NET G3, similar outcomes have been reported for chemotherapy, both for CAPTEM (RR 23%–41%, PFS 5.7–14.1 months and OS 31.7–41.2 m) and FOLFOX (RR 25%–56% and PFS 6.9–16.5 months).^24^, ^25^, ^26^, ^27^, ^28^, ^29^, ^30^, ^31^ Most studies have included patients that received CAPTEM in different treatment lines. In a retrospective first‐line study that included 22 patients with NET G3 given CAPTEM, the PFS was 12 months.^25^ In a prospective phase II study with centralised pathological re‐evaluation, temozolomide/everolimus was given to 26 patients with digestive NET G3 as first‐line treatment. The RR was 27%, PFS 12.6 months and OS 31.4 months.^31^ The optimal duration of temozolomide‐based chemotherapy is debated. Based on anatomical considerations, many clinicians prefer first‐line FOLFOX for small intestinal/colon primaries, but data are lacking to support such a treatment strategy. Based on the effect in NET G1‐2, streptozotocin/5‐FU could be considered for pancreatic primaries, but specific data for NET G3 are limited.^25^

Platinum/etoposide seems to be an inferior first‐line treatment for most NET G3, as RRs are low (0%–24%) and PFS (2.4–5 months) and OS (14 months) short.^24^, ^30^ In the Nordic NEC 2 study, first‐line platinum/etoposide treatment led to a significantly shorter PFS and OS compared to first‐line temozolomide‐based treatment in patients with NET G3 with a Ki‐67 < 55%.^7^ This study illustrates that when using first‐line chemotherapy for NET G3 with a Ki‐67 < 55%, the best chemotherapy schedule must be used up front, as later lines of treatment do not compensate for a suboptimal first‐line chemotherapy option. It also emphasised the importance of the initial pathological work‐up separating NET G3 from NEC, which can be difficult even among NET experts.^24^ For NET G3 cases with a Ki‐67 > 55% or with rapid tumour growth, platinum/etoposide may be an appropriate option.^51^

There are very limited data on the use of SSAs in patients with NET G3 except for the NETTER‐2 data,^35^ and its use in NET G3 is likely best restricted to those with hormonal hypersecretion.

### Selection of first‐line treatment

PRRT is now a first‐line treatment option for patients with NET G3 with homogeneous SRI uptake (best determined by dual PET). However, it is uncertain if it should be the first‐line standard treatment for all patients with NET G3. The SSA comparator arm in NETTER‐2 is not clinically used in everyday practice as first‐line treatment for patients with NET G3. Furthermore, there are no randomised data comparing PRRT with chemotherapy, which has been the most frequently used first‐line treatment option for patients with NET G3. In relation to sequencing, it has not been established if there is a survival benefit of giving PRRT to patients with well‐differentiated NET as a first‐line option, compared to giving it in later lines. However, the level of evidence supporting first‐line PRRT (randomised phase III trial subset analysis) is stronger than for chemotherapy; although the number of NET G3 cases in NETTER‐2 is limited (*N* = 79, 35%). Although the PFS in NETTER‐2 (22.2 months) seems numerically much better compared to first‐line chemotherapy (7–13 months), such a cross‐study comparison has many biases. This includes the fact that SRI‐positive tumours have a better prognosis, and the SSA control arm may have induced a selection bias towards patients with lower tumour bulk or less aggressive disease than the average patient population with NET G3 that is generally given chemotherapy. Results from the COMPOSE study (NCT04919226) (Table 3) in high‐grade NET G2 and NET G3 comparing first‐line PRRT with ^177^Lu‐edotreotide versus FOLFOX or CAPTEM or everolimus will provide more relevant data to help the selection of first‐line treatment for patients with advanced NET G3. A possible role for the DNA repair enzyme O6‐methylguanine‐DNA methyltransferase (MGMT) screening in treatment selection for alkylating‐based chemotherapy needs further investigation.^52^

**TABLE 3:** Selected listed studies on Clinicaltrials.gov including patients with advanced digestive Grade 3 well‐differentiated neuroendocrine tumours.

NCT number	Phase	Population	Planned enrolment	Primary end‐point (s)	Location
04365023	Epidemiological (observational)	Well‐differentiated Grade 3 digestive NET	168	OS (on first‐line platinum‐based vs. non‐platinum‐based chemotherapy)	France
04977596	Observational	Establishing a diagnostic model for Grade 3 pancreatic NETs and comparing survival against pancreatic ductal adenocarcinoma	78	Tumour characteristics	China
06889493	Phase 1	Seneca Valley Virus‐001 (SVV‐001) with nivolumab and ipilimumab in poorly differentiated NEC or well‐differentiated high‐grade NET	36	Maximum tolerated dose/recommended phase 2 dose/toxicity	USA
04525638	Single‐arm phase 2	^177^Lu‐Dotatate & nivolumab in Grade 3 NET or NEC	30	ORR (RECIST 1.1) at week 15 (+/− 1 week)	Spain
05627427	Single arm phase 2	Surufatinib & sintilimab in metastatic NENs and pancreatic carcinoma who failed standard chemotherapy	60	PFS	China
03278379 (NET‐002)	Single arm phase 2	Avelumab in unresectable or metastatic progressive Grade 2–3 well‐differentiated NET	17	ORR	Canada
03079440	Single arm phase 2	Temozolomide/capecitabine in Grade 3 and low Ki‐67 gastroenteropancreatic NETs	31	PFS	Korea
04919226 (COMPOSE)	Randomised phase 3	^177^Lu‐Edotreotide as 1st or 2nd line vs. standard of care in well‐differentiated aggressive Grade 2 or 3 SSTR + NET of gastroenteric or pancreatic origin	250	PFS	USA, Australia, Europe, India

*Note*: Clinicaltrials.gov last accessed on May 21st 2025.

Abbreviations: NEC, neuroendocrine carcinoma; NENs, neuroendocrine neoplasms; NET, neuroendocrine tumour; ORR, overall response rate; OS, overall survival; PFS, progression‐free survival; RECIST, Response Evaluation Criteria in Solid Tumours; SSTR, somatostatin receptor‐positive.

Issues to consider when selecting first‐line treatment for patients with NET G3 include Ki‐67 (chemotherapy may be more appropriate for Ki‐67 >30%–40%), tumour growth rate (slower growth rate favours PRRT), primary site (pancreatic NET more responsive to chemotherapy), time to response (likely quicker with chemotherapy) and symptom burden (rapid access to chemotherapy). An added issue in Europe is the current lack of *European Medicine Agency* approval for PRRT in patients with advanced NET G3.^53^

Specific PRRT data for NET G3 with a Ki‐67 >55% are lacking, as NETTER‐2 excluded these patients. These patients should probably not be given PRRT as first‐line treatment, as these tumours usually demonstrate aggressive clinical behaviour, more consistent with a NEC and may thus be more likely to respond to a similar management strategy. For patients with SSTR‐negative disease and Ki‐67 <55%, initial chemotherapy should be considered.

3b. What treatment should be given to patients with digestive NET G3 in the post first‐line advanced setting?

The optimal approach to later‐line therapy for advanced digestive NET G3 is not established, as there are limited data to support one sequence of therapy over another. Clinical trial enrolment should be considered, if possible. Treatment should be individualised, and patients should generally be treated with regimens that have not been received previously. If progression on first‐line treatment with chemotherapy is documented, PRRT should be considered as the next treatment, provided tumours show homogeneous SRI uptake. Further treatment with another chemotherapy regimen is another possible option. If PRRT has been given as first‐line, chemotherapy should be considered as the next option. Re‐treatment with PRRT could be a possible option, although data are lacking.

### Medical treatment

For patients who progress after PRRT, chemotherapy (CAPTEM, FOLFOX or Streptozotocin/5‐FU) should be considered rather than other systemic treatment options. There is no current evidence to support the use of 5‐FU/irinotecan (FOLFIRI), but extrapolating from NEC,^54^, ^55^ it may be an option beyond FOLFOX. Several small studies support a role for targeted therapy, particularly for NET G3 with a Ki‐67 index in the range of 20%–30%; the use of targeted therapy is discussed later in this manuscript (Controversy 5: “Treatment of advanced disease with targeted therapy”).

There are no data to support the use of immune checkpoint inhibitors (ICIs) in unselected patients with advanced digestive NET G3. This is dealt with in a later section of this manuscript entitled (Controversy 4: “Treatment of advanced disease with immunotherapy”).

A NET G3 diagnosis can become more aggressive over time, lose SSTR expression, and, in rare cases, transform to a NEC‐like genetic profile.^13^ Such patients should likely be treated similarly to those with a NEC diagnosis. However, their prognosis seems to be extremely poor.^16^

### PRRT

Retrospective studies indicate that PRRT given in later lines has a benefit in high‐grade digestive NEN.^56^ In three studies, separate data for the NET G3 subgroup were provided. One study evaluated PRRT primarily as second‐line treatment (42%) or beyond (38%) in 149 patients with high‐grade digestive NEN.^57^ The study included 60 NET G3 cases given PRRT with a RR of 42%, PFS 19 months, and OS 44 months. Other later‐line studies (*n* = 19) have reported RRs of 28%–38% and PFS of 12.9–13.1 months after PRRT for patients with NET G3.^58^ In another small study (*n* = 20), patients with NET with a Ki‐67 15%–55% treated with PRRT and a radiosensitiser (CAPTEM) had better outcomes compared to PRRT alone: RR 70% vs. 20%, PFS 26 months vs. 12 months, and OS was not reached vs. 51 months.^59^ However, the limited number of patients included in this study precludes definitive conclusions.

**FIGURE 1:**
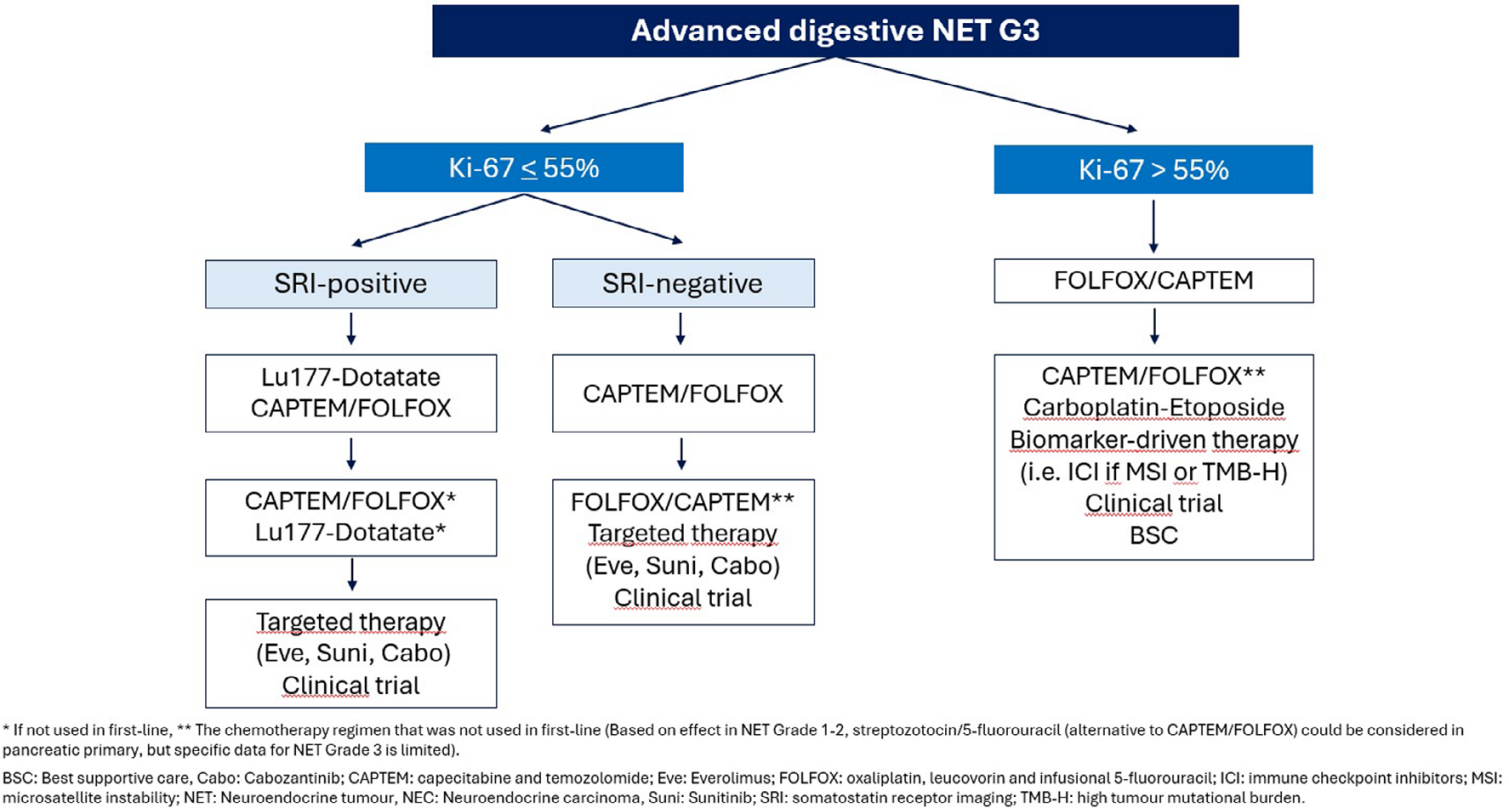
Suggested algorithm for treatment of patients with digestive G3 well‐differentiated NET.

A suggested algorithm for treatment of patients with digestive well‐differentiated NET G3 is provided in Figure 1.

4 Treatment of advanced disease with immunotherapy:

4a. Is there a role for immunotherapy in the treatment of patients with advanced digestive NET G3?

The role of immunotherapy in the treatment of patients with high‐grade NET is unclear. A number of small prospective trials have investigated the potential benefit of treating this cohort of patients with single‐agent immunotherapy or in combination, based on reported rates of programmed death‐ligand 1 (PD‐L1) expression (ranging from 14% to 50%) and relatively high mutation load.^60^ In addition, several drugs are *Food and Drug Administration* (FDA)‐approved for tumour‐agnostic indications that may occur in NET G3, such as pembrolizumab, which is approved for microsatellite instability (MSI)/deficient mismatch repair (dMMR) and Tumour Mutation Burden (TMB)‐high tumours, and dostarlimab, which is approved for MSI/dMMR tumours, although these molecular features are uncommon in this setting.^3^ Some of these studies will now be discussed, but none focused specifically on NET G3, and central pathology review was not always performed.

In a combined analysis of two prospective, non‐randomised trials with pembrolizumab monotherapy in patients with previously treated metastatic high‐grade NEN (*N* = 29; 24 (83%) had either a pancreas or gastrointestinal primary, and 9 (31%) had well‐differentiated morphology), a disease control rate of 24.1% was reported.^61^ There was no difference in outcomes based on PD‐L1 expression.

Another combined analysis study reported on the efficacy and safety of the PD‐L1‐directed antibody avelumab as monotherapy in patients with advanced progressive G 2–3 NEN.^62^ There were 27 patients included in this study, with 6 (22%) having well‐differentiated G3 morphology. The disease control rate at 6 months was 21%, with a median PFS of 3.3 months and a median OS of 14.2 months. Both studies^61^, ^62^ concluded that single‐agent immunotherapy in these patient cohorts had limited activity; further research, especially on combination strategies, was recommended.

Toripalimab was also assessed in pretreated patients with G2‐3 NEN (Ki‐67 > 10%), mostly of GEP origin, including 32 NEC and 8 NET.^63^ They reported a RR of 25% for the small NET subgroup and, interestingly, a significantly higher RR in patients with TMB‐high or PD‐L1 expression ≥10% compared to those that had TMB‐low tumours (75.0% vs. 16.1%, *p* = 0.03) or PD‐L1 < 10% (50.0% vs. 10.7%, *p* = 0.019), while 3 of the 8 (37.5%) responders harboured *ARID1A* mutations.

In the SWOG S1609 phase II basket trial of nivolumab in combination with ipilimumab in patients with previously treated high grade NEN (rectum, gastro‐oesophageal junction, cervix, pancreas and unknown primaries), a RR of 26% was reported. However, of 19 patients included, only one patient had well differentiated morphology, one had moderate differentiation, while in 6 patients, differentiation was unknown, with the remaining having poorly differentiated morphology; therefore, the benefit in NET G3 is unclear.^64^ The efficacy of nivolumab in combination with ipilimumab was also assessed in a retrospective study of patients with previously treated high grade NEN.^65^ Seven (20.6%) of 34 patients had NET G3. Primary sites included gastrointestinal, gynaecological, and unknown origin. The RR was 14.7%, disease control rate 41.2%, PFS 1 month, and OS 5 months. Unfortunately, this combination had only modest activity in this aggressive and heavily pretreated population.

The combination of targeted therapy with immunotherapy may be an avenue to explore in this patient population.^66^ Regorafenib (oral multi‐kinase inhibitor) was combined with avelumab in a phase II trial including patients with previously treated advanced GEP NEN.^67^ In the 8 patients with NET G3, 4 (50%) had stable disease and the other 4 had progressive disease. The Bayesian mean posteriors RR for these 8 patients was 10% (95% confidence interval 0%–34%). Although the findings in the entire cohort of a 6‐month RR of 18%, with a median PFS of 5.5 months, were considered to have potential, it was highlighted that predictive markers and validation in randomised clinical trials were needed.

In 2020, the FDA granted the second pembrolizumab tumour‐agnostic indication for the treatment of advanced TMB‐high (≥10 mut/Mb) solid tumours based on the KEYNOTE‐158 trial.^68^ Among the 805 patients enrolled who were evaluable for TMB, 105 (13%) were TMB‐high, including 5 of 87 (5.7%) patients with neuroendocrine tumours. However, tumour site, differentiation, and grade were not specified. The RR was 28% among these 81 patients with non‐MSI TMB‐high tumours. Two of the 5 (40%) patients with TMB‐high NET achieved a RR. The median duration of response was not reached, and 50% of patients had a response duration greater than 2 years. The precise incidence of TMB‐high in digestive NET G3 is, however, uncertain.

Induction chemotherapy could have the potential of increasing TMB prior to immunotherapy administration and increasing its efficacy in this patient population.^66^ Strosberg et al.^69^ reported on the efficacy of immune checkpoint inhibitors in patients with advanced pancreatic NET displaying high TMB and MMR alterations following treatment with alkylating agents. Thirty‐nine patients were included in this retrospective study (42% had G3 disease). The overall RR was 18% with a disease control rate of 46%, with a median PFS of 2.8 months. Patients with high TMB and altered MMR had higher RRs (30% vs. 0%, *p* = 0.03 and 42% vs. 7%, *p* = 0.02, respectively). It was concluded that immunotherapy had limited efficacy in unselected patients with pancreatic NETs pretreated with alkylating agents, but that it may be an option for cases with high TMB and MMR alterations.^69^

To date, there is no role for the routine clinical use of immunotherapy in the treatment of patients with advanced digestive NET G3 outside of clinical trials except for the exceptional cases with high TMB or MMR alterations; therefore, testing for these biomarkers is recommended, where possible.

5 Treatment of advanced disease with targeted therapy:

5a. Is there a role for targeted therapy in the treatment of patients with advanced digestive NET G3?

Tyrosine kinase and mTOR inhibitors (sunitinib and everolimus, respectively) have been used in the treatment of mainly G1/2 pancreatic and extra‐pancreatic NET (epNET); although the pivotal trials included well‐differentiated tumours, the G3 subgroup was not excluded. However, the proportion of patients with NET G3 included in these trials is low, and efficacy has not been specifically reported for this subgroup. In a retrospective study where 10 patients with pancreatic NET G3 (median Ki‐67 35%) received sunitinib, disease control was reported in 9, with a PFS of approximately 6 months.^34^ In a prospective phase II trial, 6 patients with well‐differentiated (WD) pancreatic NET and 6 with poorly‐differentiated (PD) NEC (but with Ki‐67 values between 20% and 50%) received sunitinib following disease progression.^70^ Within the WD‐NET cohort, 4/6 had a similar response to PD‐NEN with a Ki‐67 <50%; sunitinib exhibited the highest activity in NEN G3 with a Ki‐67 cut‐off value of 25%.^70^ A phase II trial with pazopanib in 13 patients with NET G3 (pancreatic and colorectal) reported a RR of 23% and a median PFS of 5.8 months.^71^ A retrospective subgroup analysis of the 24 patients with NET G3 (12 pancreatic and 12 epNETs) included in the CABINET randomised trial reported an increased RR (25% vs. 0%) and PFS for patients treated with cabozantinib versus placebo, particularly in the pancreatic NET G3 subgroup (pancreatic: 13.5 vs. 1.5 months, *p* = 0.0004; epNETs: 6.5 vs. 4.2 months, *p* = 0.15), consistent with the global trial results.^72^, ^73^ Cabozantinib was also evaluated in a single‐arm phase II trial (CABONEN) of 32 patients with advanced, mainly digestive NET G3 with a Ki‐67 of <60%.^74^ In preliminary results, cabozantinib demonstrated a 64% disease control rate at 6 months and a PFS of 7 months. Although surufatinib showed activity following disease progression in both patients with epNET and pancreatic NET with a Ki‐67 >10%, disease‐specific activity in NET G3 was not reported.^75^, ^76^ In a retrospective analysis of everolimus, 15 patients with pancreatic NET (4 first‐line and 11 following chemotherapy), who had WD morphology and a Ki‐67 <55% (median Ki‐67 30%), had a median PFS of 6 months and OS of 28 months. Six patients maintained a response for at least 12 months.^77^ Although it appears that these agents may be active in some patients with NET G3 following disease progression, there is insufficient evidence, to date, to guide patient selection.

6 Other controversies:

6a. Is there a need for re‐biopsy to guide treatment decision making in patients with digestive Grade 1–Grade 3 well‐differentiated NET?

Neuroendocrine tumours and NEC are considered distinct tumour entities with unique genetic profiles. NEC typically exhibits genetic abnormalities that resemble their exocrine counterparts, such as *TP53, KRAS, BRAF*, or Rb1 genes co‐alterations in *TP53* and *RB1*, whereas the genetic alterations in NET are site‐specific. For example, pancreatic NET frequently has somatic mutations in *MEN1, ATRX, DAXX*, and *TSC1/2*,^78^ whereas small intestinal NET (siNET) shows predominantly chromosome 18 loss and sometimes *CDKN1B* mutations.^79^

In 2024, Joseph et al.^12^ provided insights into the biological and genetic progression of rapidly advancing and lethal metastatic NET G3 that originated from lower‐grade NET. Their study described five patients initially diagnosed with G1/G2 or lower‐proliferative NET. In all cases, *RB1* and *TP53* alterations were synchronously acquired in the metastatic high‐grade tumours. Despite the high‐grade transformation, these tumours retained the molecular alterations present in the corresponding lower‐grade components. The Ki‐67 indices ranged from 60% to 94%. The reported median OS following the high‐grade transformation, characterised by *RB1* loss and aberrant TP53 expression, was 12 months.^12^

Backman et al.^14^ reported that alkylating chemotherapy given to patients with G1/G2 pancreatic NETs was linked to the development of MMR deficiency, a DNA hypermutational phenotype, and progression to high‐grade NEC and metastases in a subset of tumours. The study proposed that DNA‐damaging therapies may drive tumour evolution, increasing mutagenesis and facilitating high‐grade transformation in a subset of G1/G2 NET.

A recent prospective study has looked at the impact of re‐characterising progressive pancreatic NETs (including clinical, biochemistry, re‐biopsy, and imaging).^15^ Of 21 patients, 16 underwent re‐biopsy, and 81.3% had an increase in Ki‐67, with transition from G2 to G3 in 50%, leading to therapy change in some, emphasising the need to re‐evaluate all relevant aspects of disease in these patients.

However, there is also emerging evidence suggesting that some NET may progress to NEC in the gastrointestinal tract. Recent studies have highlighted the evolving classification challenges surrounding a subset of NET G3. These challenges arise from their overlapping morphological characteristics and/or the acquisition of NEC‐like molecular alterations during tumour progression, especially in the context of multi‐modal systemic therapies.^12^, ^13^, ^14^

In 2024, Kasajima et al.^13^ identified acquired *TP53* mutations and NEC‐like histological features (e.g., high‐grade atypia, diffuse growth patterns, and necrosis) in a subset of metastatic G3–Gastroenteropancreatic NETs associated with rapid tumour progression. These metastatic NET G3 originated from tumours without NEC‐like features and a median Ki‐67 labelling index of 10%, which increased to a median of 65% during progression. The authors termed these aggressive, rapidly progressing high‐grade NEN as “NEC‐like NETs.” Despite their aggressive behaviour and the prevalence of *TP53* alterations, most retained molecular features characteristic of NET, with only one NEC‐like NET acquiring an *RB1* alteration during disease progression.

Due to these findings as well as the differing biological behaviours and treatment responses that exist between NET G1‐2 and NET G3 and between NET G3 and NEC,^78^ re‐biopsy of gastro‐enteropancreatic NET is recommended to guide treatment decisions when there is a shift in the progression rate, evidence of heterogeneity in disease progression, a discrepancy between morphological and molecular imaging findings, or when the clinical situation deviates from the usual seen.

6b. Is Grade 3 well‐differentiated NET outside of the digestive tract treated similarly?

Tumours corresponding to NET G3 have been identified in the lung, showing similar morphogenetic features that have been observed in NET G3 of the digestive tract.^5^, ^80^, ^81^ So far, it is not known how these likely NET G3 of the lung compare with the lung NENs discussed below. There is also no definitive guidance on the treatment of pulmonary tumours which have been diagnosed as NET G3. In 2019, Alcala et al.^82^ identified six samples with low‐grade lung NET morphology but with molecular and clinical characteristics closely resembling high‐grade lung NEC. These tumours, termed “supra‐carcinoids,” exhibited transcriptomic and epigenetic profiles indistinguishable from those of lung NEC.^82^ The discovery of these aggressive supra‐carcinoids, whose existence has also been validated in other studies,^83^, ^84^, ^85^ underscores a significant clinical gap in identifying patients with NET who have a poorer prognosis and therefore have unique treatment requirements.

There is evidence to suggest that NET may progress toward a more aggressive, NEC‐like molecular profile in the lung as well. In 2025, Rekhtman et al.^86^ performed an in‐depth clinicopathologic, genomic, and transcriptomic analysis of a subset of small‐cell lung cancers (SCLCs) that, despite their aggressive clinical behaviour, displayed genomic and pathological similarities with carcinoids. These tumours accounted for half of the SCLC cases observed in never or light smokers. Notably, they lacked the typical *TP53* and *RB1* co‐inactivation but exhibited chromothripsis—a phenomenon of extensive localised chromosome fragmentation—resulting in extra‐chromosomal amplification of *CCND1* or co‐amplification of *CCND2/CDK4/MDM2*. These amplifications are known to functionally inactivate *RB1* and *TP53* proteins, respectively. The authors proposed that the findings suggest a previously unrecognised pathway of lung NEC development in never smokers, possibly through transformation from lower‐grade lung NETs or their progenitors.^86^ The recommended treatment for these entities is uncertain and tends to follow that of NET G3 of the digestive tract.^87^

## 3 SUMMARY

The available data to guide treatment decision‐making for patients with digestive NET G3 are sparse. Multidisciplinary discussion and management are of paramount importance, as are multi‐institutional collaborative biobanks and registries. Surgery or localised therapies should be considered where appropriate. In the adjuvant and advanced setting, clinical trial enrolment is favoured, if possible. Temozolomide with capecitabine is a first‐line advanced treatment option in patients with a Ki‐67 ≤55%, with PRRT an alternative option in SRI‐positive cases. 5‐fluorouracil‐based therapy (5‐fluorouracil/oxaliplatin) is recommended in the second‐line advanced setting. The use of targeted therapy (everolimus, sunitinib, cabozantinib) may be an option in selected cases. Immunotherapy is currently not recommended outside of the approved tumour‐agnostic indications, that is, for pretreated MSI/dMMR or TMB‐high tumours. There are ongoing studies in the adjuvant (Table 2) and advanced setting investigating imaging modalities, epidemiology and treatment, including chemotherapy, radioligand therapy, targeted therapy and immunotherapy (Table 3), which may further inform and educate the treating community. Randomised international studies with translational correlates are necessary and favoured.

## AUTHOR CONTRIBUTIONS

**Mairéad G. McNamara:** Conceptualization; writing – original draft; methodology; writing – review and editing; project administration; supervision; data curation; investigation. **Halfdan Sorbye:** Conceptualization; writing – original draft; methodology; writing – review and editing; investigation. **Nehara Begum:** Conceptualization; investigation; writing – original draft; methodology; writing – review and editing. **Emanuel Christ:** Conceptualization; investigation; writing – original draft; methodology; writing – review and editing. **Nicola Fazio:** Conceptualization; writing – original draft; methodology; writing – review and editing; investigation. **Lynnette Fernandez‐Cuesta:** Conceptualization; investigation; writing – original draft; methodology; writing – review and editing. **Rocio Garcia‐Carbonero:** Conceptualization; investigation; writing – original draft; methodology; writing – review and editing. **Gregory Kaltsas:** Conceptualization; investigation; writing – original draft; methodology; writing – review and editing. **Atsuko Kasajima:** Conceptualization; investigation; writing – original draft; methodology; writing – review and editing. **Ramon Salazar:** Conceptualization; investigation; writing – original draft; methodology; writing – review and editing. **Ernst Jan M. Speel:** Conceptualization; investigation; writing – original draft; methodology; writing – review and editing. **Andreas Kjaer:** Conceptualization; investigation; writing – original draft; methodology; writing – review and editing.

## CONFLICT OF INTEREST STATEMENT

Mairéad G. McNamara: received research grant support from Astra Zeneca, Servier, Ipsen, and NuCana. She has received travel and accommodation support from Bayer and Ipsen, and speaker honoraria from Pfizer, Ipsen, NuCana, Mylan, and AAA. She has served on advisory boards for Celgene, Ipsen, Sirtex, Baxalta, Incyte, and Astra Zeneca. Halfdan Sorbye: Advisory board for BMS, Hutchinson, Novartis, and Ibsen. Speaker honoraria from Ipsen, Novartis, SAM Nordic, Pierre Fabre, Daichi Sanchi. Nehara Begum: Advisory board for ESTEVE and Ipsen Pharma, speaker honoraria from ESTEVE, Ipsen Pharma, and Recordati. Emanuel Christ: received research grant support from Ipsen, Pfizer, and Novartis. He has received travel and accommodation support from Ipsen and speaker honoraria from Pfizer and Ipsen. She has served on advisory boards for Ipsen, Pfizer, and Novartis. Nicola Fazio: Astellas, Advisory Board, Personal, Astellas, Invited Speaker, Personal, ASTRA ZENECA, Other, Personal, Steering Committee, AstraZeneca, Advisory Board, Personal, ESTEVE, Advisory Board, Personal, IPSEN, Advisory Board, Personal, IPSEN, Other, Personal, Steering committee, ITM, Advisory Board, Personal, Merck, Advisory Board, Personal, NOVARTIS, Invited Speaker, Personal, NOVARTIS, Advisory Board, Personal, Astellas, Local PI, Institutional, Financial interest, IPSEN, Local PI, Institutional, Financial interest, IPSEN, Research Grant, Institutional, Financial interest, ITM, Local PI, Institutional, Financial interest, Merck, Research Grant, Institutional, Financial interest, MSD, Local PI, Institutional, Financial interest, NOVARTIS, Research Grant, Institutional, Financial interest, NOVARTIS, Local PI, Institutional, Financial interest, Revolution medicine, Local PI, Institutional, Financial interest. AIOM, Other, Internal reviewer of NET guidelines, ENETS, Other, President elect, ESMO, Other, Member of the NET Faculty, ITANET, Other, Member of the scientific board, SPARC Europe, Other, Steering committee. Lynnette Fernandez‐Cuesta: Where authors are identified as personnel of the International Agency for Research on Cancer/World Health Organization, the authors alone are responsible for the views expressed in this article; they do not necessarily represent the decisions, policy, or views of the International Agency for Research on Cancer/World Health Organization. Rocio Garcia‐Carbonero has received honoraria for speaker engagements, advisory roles, or funding for continuous medical education from AAA‐Novartis, Advanz Pharma, Astellas, Bayer, BMS, Boehringer, Crinetics, Esteve, GSK, Hutchmed, Ipsen, ITM, MSD, PharmaMar, Pierre Fabre, Sanofi, Servier, and Takeda. Gregory Kaltsas: No conflicts to declare. Atsuko Kasajima: Advisory board for Ipsen and Boehringer‐Ingelheim. Ramon Salazar: Advisory board for Takeda, WNT Pharma, speaker honoraria from Esteve Pharma and Novartis, spouse owner of SACE Medhealth until December 2023 (commercial medical education company). Ernst Jan M. Speel: No conflicts to declare. Andreas Kjaer: Is an inventor of 64Cu‐DOTATATE (SRI). Advisor Novo Nordisk A/S. Speaker honoraria from Curium.

## Data Availability

Data sharing not applicable to this article as no datasets were generated or analysed during the current study.
